# Damages of the tibial post in constrained total knee prostheses in the early postoperative course – a scanning electron microscopic study of polyethylene inlays

**DOI:** 10.1186/1471-2474-9-83

**Published:** 2008-06-11

**Authors:** Adrian Skwara, Carsten O Tibesku, Rudolf Reichelt, Susanne Fuchs-Winkelmann

**Affiliations:** 1 Department of Orthopaedics, University Hospital Marburg, University of Marburg, Baldingerstrasse, D-35041 Marburg, Germany; 2 Orthopädisch-chirurgische Gemeinschaftspraxis, sporthopaedicum Straubing, Bahnhofplatz 8, D-94315 Straubing, Germany; 3 Institute for Medical Physics and Biophysics, University Hospital Muenster, University of Muenster, Robert-Koch-Str. 31, D-48149 Muenster, Germany

## Abstract

**Background:**

Investigation of the risk of fracture of the polyethylene (PE) inlay in constrained total knee prostheses.

**Methods:**

Three unused and seven polyethylene inlays that had been implanted in a patient's knee for an average of 25.4 months (min 1.1 months, max 50.2 months) were investigated using scanning electron microscopy (SEM). All inlays were of the same type and size (Genesis II constrained, Smith & Nephew). The PE surface at the transition from the plateau to the post was analyzed.

**Results:**

The unused inlays had fissure-free surfaces. All inlays that had been implanted in a patient's knee already had distinct fissures at the front and backside of the post.

**Conclusion:**

The fissures of the transition from the plateau to the post indicated a loading-induced irreversible mechanical deformation and possibly cause the fracture of the inlay.

## Background

Due to the growing number of revision total knee arthroplasties, posterior stabilized and constrained total knee prostheses have become more and more popular in recent years. They allow intrinsic stabilization in knees with ligamentous instability. So far, clinical results of constrained total knee arthroplasty (TKA) have been reported only in medium-term follow-up [1]. Nevertheless, hinged prostheses are still being discussed for salvage total knee arthroplasty [2]. However, if stability can not be obtained with an unconstrained implant progressive levels of constraint, but as little constraint as possible should be used [3].

Posterior stabilized total knee prostheses that are similar in design to constrained prostheses also tend to increasing axis deviations and inlay breakage after a few years, especially in patients with severe preoperative axis deviation of the leg axis of more than 10° in the coronal plane [4]. Several case reports described a fracture of the polyethylene tibial post in different posterior stabilized knee prostheses even if the tibial post was reinforced by a metal rod [4-7]. Studies about retrieved posterior stabilized knee prostheses showed that especially the backside of the post can be a source of polyethylene wear [4,8]. Li et al. demonstrated in a cadaver study after TKA a higher contact force at the tibial post and less posterior femoral translation at low flexion and hyperextension resulting an anterior post impingement and additional polyethylene wear [9]. In unconstrained flat-on-flat total knee prostheses a correlation between the patients' activity and the creep reaction and deformation of the polyethylene was reported [10]. Other parameters, such as the kind of sterilization, manufacturing and thickness of the inlay have been pointed out repeatedly and have been optimized by many manufacturers worldwide.

Due to the increasing deviation of the mechanical leg axis and breakage of the post, the post has to be regarded as the weak point of the constrained total knee joint arthroplasty, where the tibial post is not reinforced with a metal rod. From the mechanical point of view, however, the transition between the post and the femoral cam cause extensive loads on the post and occurs as a weak point of this design. This proved to be a serious problem with constraint prostheses, which are expected to provide a higher stability and tolerate these acting forces. The following investigation of unloaded and loaded constraint polyethylene inlays was performed to elucidate initially this problem in polyethylene inlays without metal rod reinforcement.

## Methods

Ten polyethylene inlays were investigated using scanning electron microscopy. All inlays were of the same type and size. The model used was an 11 mm thick constrained inlay of the Genesis II total knee (Genesis II constraint, Smith & Nephew, Schenefeld, Germany). The size is called "5–6" which is identical for sizes 5 and 6 of the tibial component. The inlays consist exclusively of ultrahigh-molecular weight polyethylene (UHMWPE; ASTM F 648) without any metal reinforcement and were formed by milling to its final shape. All inlays were sterilized by gas sterilization using ethylene oxide.

Three polyethylene inlays were unused and acted as controls. The samples were subsequently prepared according to a standardized preparation protocol, mentioned below.

Seven inlays had been retrieved from patients, four men and three women, with a constrained prosthesis during revision surgery. The mean age of the patients was 66.4 years (min 48.6 years, max 80.0 years). The patients had a body mass index at the time of surgery of 31 kg/cm^2 ^(min 24.2 kg/cm^2^, max 38.6 kg/cm^2^).

All UHMWPE inlays were retrieved during second revision TKA. The first revision was carried out for aseptic loosening in four cases, septic loosening in one case, and mediolateral instability in two cases. In this first revision, a condylar constrained implant was used for reconstruction. After a period of in average 25.4 months after the first revision (min 1.1 months, max 50.2 months), a second revision was necessary. The reasons for the second revision were aseptic loosening in three cases, deep infection in two cases and in two cases a painful combined medial and lateral instability. Preoperatively, the patients showed no significant deviation of the mechanical axis in coronal plane and had no trauma in their history.

The retrieved implants were cleaned with sterile water and afterwards prepared according to the same preparation protocol applied to the other inlays.

**Figure 1:**
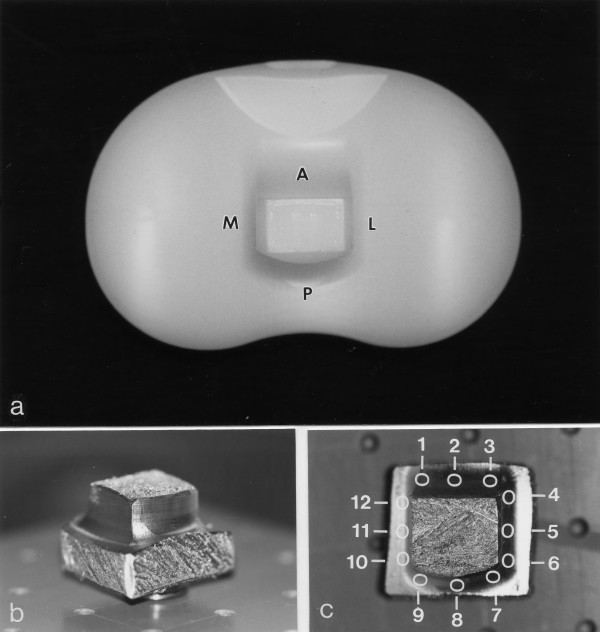
**Photographs of a constraint UHMWPE prosthesis**: (a) Different sides of the post are marked as follows: A: anterior, L: lateral, M: medial, P: posterior. (b) Sawed off UHMWPE prosthesis mounted on an aluminum specimen stub with electrically conductive carbon and sputtered with 15 nm gold for scanning electron microscopic investigation. (c) Top view to the prosthesis with the marked specific locations defined by the running numbers 1 to 12.

The polyethylene inlays, which have an overall size of about 75 mm mediolateral, 50 mm posteroanterior and a height of 36 mm (Fig. 1a), had to be reduced in size for the scanning electron microscopic investigation. To fulfill the instrumental requirements the outer regions of the UHMWPE prostheses were sawed off very carefully using a special saw with fine saw teeth. The final size of the prostheses after sawing amounts to approximately 26 mm × 26 mm × 20 mm (Fig. 1b). The prostheses were cleaned twice in 96% ethanol applying ultrasound each time for 5 minutes. Subsequently, the cleaned prostheses were mounted on aluminum specimen stubs with electrically conductive carbon (PLANO, Wetzlar, Germany) and sputter coated with gold using argon gas as the ionizing plasma. The average thickness of the gold film applied to the prostheses was approximately 15 nm.

Imaging was performed on a scanning electron microscope (SEM) S-450 (Hitachi Ltd., Japan) with secondary electrons (SE) at 20 keV and at room temperature [11,12]. The primary magnifications were in the range of 50- to 3,000-times depending on whether an overview or details should be monitored. A very careful scanning electron microscopic screening of the surface structure was performed in the region where the post merges into the inlay plateau. To better compare the results obtained from different prostheses, we selected twelve specific locations (Fig. 1c) defined by their geometric positions. Micrographs were recorded from a high-resolution cathode ray tube using negative film (Agfapan, APX100). For the final demonstration of the experimental data, however, we used a total of eight different locations only, which correspond to the corner of the tibial post.

## Results

**Figure 2:**
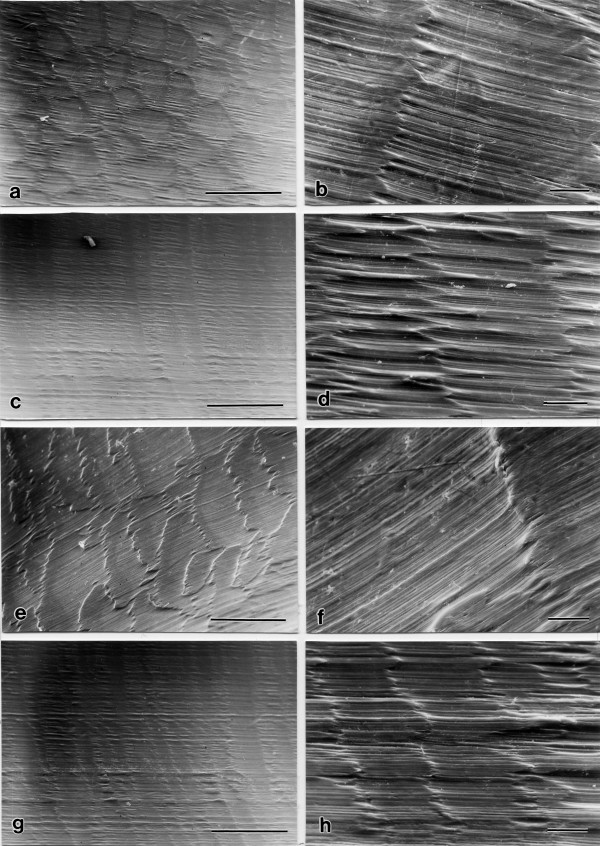
**Scanning electron micrographs of a new constraint UHMWPE prosthesis**: Recordings at different locations showing the typical cutting traces of the milling treatment. The patterns of milling traces were different at the different sides of the post but showed a very similar appearance at all locations on one side of the post: (a) Location 3, magnification 32×, bar at the bottom correspond to 500 μm. (b) Location 3, magnification 170×, bar at the bottom correspond to 50 μm. (c) Location 4, magnification 32×, bar at the bottom correspond to 500 μm. (d) Location 4, magnification 170×, bar at the bottom correspond to 50 μm. (e) Location 7, magnification 32×, bar at the bottom correspond to 500 μm. (f) Location 7, magnification 170×, bar at the bottom correspond to 50 μm. (g) Location 10, magnification 32×, bar at the bottom correspond to 500 μm. (h) Location 10, magnification 170×, bar at the bottom correspond to 50 μm.

**Figure 3:**
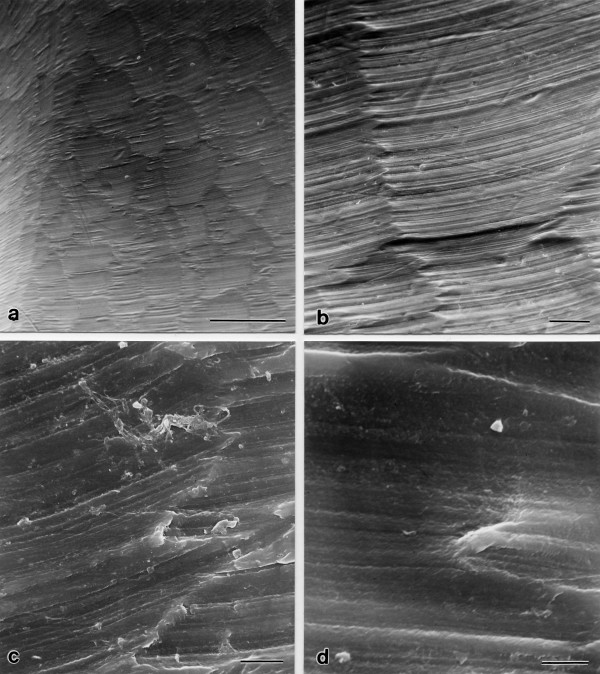
**Scanning electron micrographs of a new constraint UHMWPE prosthesis at different magnifications showing the cutting traces of the milling treatment**. (a) Location 1, magnification 32×, bar at the bottom correspond to 500 μm. (b) Location 1, magnification 170×, bar at the bottom correspond to 50 μm. (c) Location 6, magnification 900×, bar at the bottom correspond to 10 μm. (d) Location 10, magnification 1900×, bar at the bottom correspond to 5 μm.

The scanning electron microscopic investigations of the unused inlays showed typical traces and small unevenness on the surface caused by the milling-treatment during the manufacturing process. These traces occur on the entire surface area of the inlay. The SE-micrographs did not show microfissures in the material (Fig. 2 and 3). Interestingly, the patterns of milling-traces showed a very similar appearance at all locations (Fig. 2). There were no significant differences between different surface areas of the inlays.

**Figure 4:**
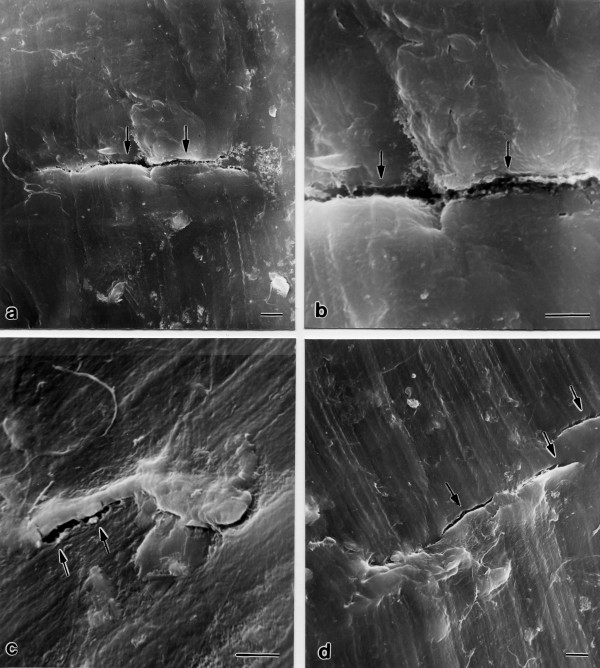
**Scanning electron micrographs of loaded constraint UHMWPE prosthesis**. Different magnifications of the UHMWPE prosthesis showing cutting traces of the milling treatment, clefts and some micro-fissures (which occur as fine black features, see arrows), and probably local material failures: (a) Location 1, magnification 900×, bar at the bottom correspond to 5 μm. (b) Location 1, magnification 1900×, bar at the bottom correspond to 5 μm. (c) Location 7, magnification 1900×, bar at the bottom correspond to 5 μm. (d) Location 9, magnification 900×, bar at the bottom correspond to 5 μm

**Figure 5:**
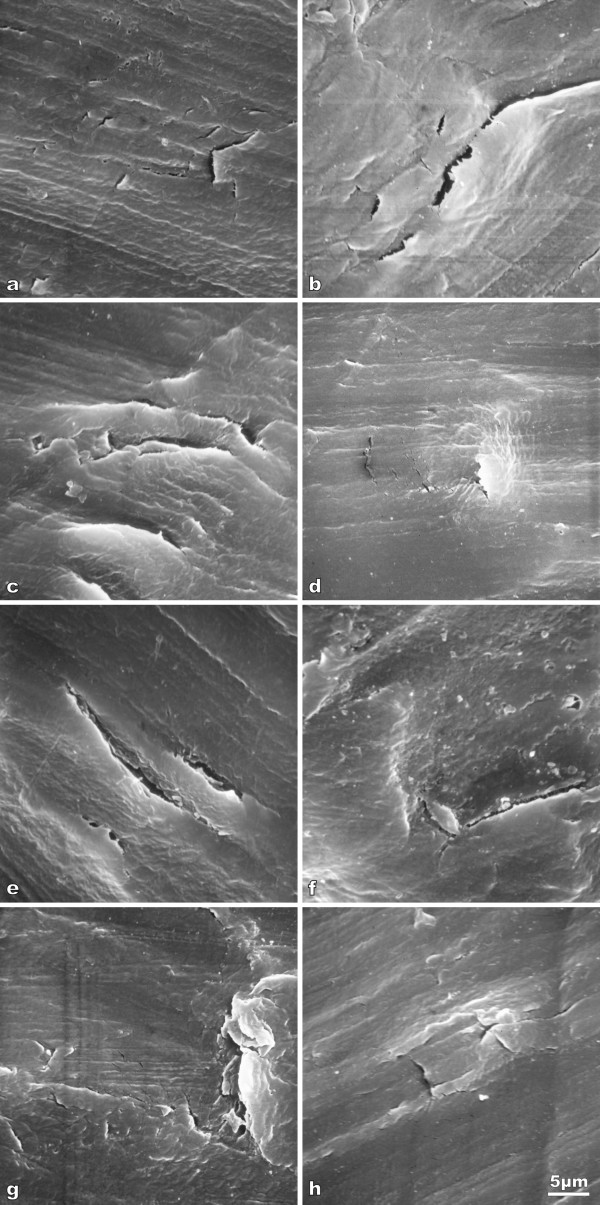
**Scanning electron micrographs of loaded UHMWPE prostheses**. The micrographs are recorded near the corners of the tibial post and show the typical cutting traces of the milling treatment, clefts, micro-fissures (which occur as fine black features) and probably also local material failures: (a) Location 1, magnification 900×. (b) Location 3, magnification 900×, bar at the bottom of (h) correspond to 5 μm. (c) Location 4, magnification 900×, bar at the bottom of (h) correspond to 5 μm. (d) Location 6, magnification 900×, bar at the bottom of (h) correspond to 5 μm. (e) Location 7, magnification 900×, bar at the bottom of (h) correspond to 5 μm. (f) Location 9, magnification 900×, bar at the bottom of (h) correspond to 5 μm. (g) Location 10, magnification 900×, bar at the bottom of (h) correspond to 5 μm. (h) Location 12, magnification 900×, bar at the bottom correspond to 5 μm.

All inlays that had been explanted from a patient's knee showed clefts and some micro-fissures. Especially the corners or the tibial post showed polyethylene damage. The anteromedial corner corresponds to the location 12/1 (Fig. 4a and 4b, Fig 5a and 5h). The anterolateral corner corresponds to location 3/4 (Fig. 5b and 5c). The posterolateral corner corresponds to location 6/7 (Fig. 4c, Fig 5d and 5e) and the posteromedial corner corresponds to location 9 and 10 (Fig. 4d, Fig. 5f and 5g). Such damages of a removed polyethylene inlay could be found already five weeks after load bearing of an overweight patient at these typical locations corresponding to the ventral and dorsal corners of the tibial post.

## Discussion

The exemplary scanning electron microscopy investigations demonstrated significant differences between the unused and the loaded inlays in a patient's knee. These findings support the hypothesis that load-induced damaging of PE-inlays in constrained total knee prostheses preferentially occur in the transition zone where the tibial post merges into the plateau of the PE-inlay. Mainly the zones at the ventral and dorsal corners of the tibial post showed distinct serious micro-fissures and micro-clefts after load application after implantation in a short-term follow-up. Hence, it can be assumed that this region involves a certain danger of fracture. Even these micro-fissures potentially can expand to macroscopic fissures of the post or may become regions of preferential polyethylene wear. If they can act as a predetermined breaking point of the post must be evaluated in further examinations. The small number and only a qualitative analysis of examined inserts could not elaborate the main mechanism for the observed damages in our collective.

Reports already described the problem of the fracture of the tibial post in different posterior-stabilized knee arthroplasties [4,6,7]. A substantial aspect of the mentioned problem may have a kinematic origin. In the physiological knee movement, internal and external rotation of as much as 12° especially at flexion angles of 0° to 40° has been documented during level walking and stair climbing [13]. The postoperative kinematics after constrained total knee prostheses has to be considered as being unphysiologic. The Genesis II condylar constrained implant generates a rotational constraint with the consequence of rotational load acting at the tibial post. Futhermore a malrotation of the femoral and tibial component can increase torsional load and impingement on the tibial post [7,14]. In particular the anterior and posterior corners of the post are concerned. The damages on the tibial post demonstrated in our investigation occurred mainly at these locations but malrotation of the components could not be detected in this collective.

Anymore movement in the normal knee joint contained a combination of rolling and sliding of the femoral condyles on the tibial plateau [15]. Rolling of the femoral condyles plays an essential role during the initial flexion phase (0 to 20°) [16,17]. Blunn et al. assumed, that especially the cyclic sliding causes major damages to the polyethylene inlay [18]. But this mechanism causes mainly a damage at the weight bearing areas of the tibial tray and less damage on the tibial post. Even if there is a malpositioning of the femoral component in a flexed position relative to the sagittal axis of the knee, or the tibial component has excessive posterior slope, anterior impingement of the femur on the tibial post also may occur in fully extension of the knee [19]. This repetitive anterior impingement between the femoral cam and the polyethylene post during full knee extension and a posterior lift-off force in high flexion cause increased load on the tibial post with the resulting damage [5,19,20]. As well the stabilization of medial and lateral knee instability or imbalance caused by unbalanced flexion and extension gap leads to an increased stress on the tibial post [21]. This seems to be the main mechanism responsible for the damage in our specimens. In our collective two patients had an instability and three were revised for aseptic loosening were small sized instability could be expected either. This cause rotational loads as well as lateral and anteroposterior loads to the post.

Finally, other specific factors like the patient's height, weight and activity, the surgical technique considering soft tissue preservation and alignment, the design of the prosthesis, its quality, the mechanical processing of the form, the thickness, the kind of sterilization of the polyethylene inlay as well as the polyethylene itself play also an important role. Muratoglu et al. [22] could show in a knee simulator investigation significant lower wear rates of highly cross-linked polyethylene compared to conventional polyethylene using a cruciate-retaining design. Unfortunately there is no research into this topic to posterior stabilized or constrained implants particularly regarding the damage occur at the tibial post. Also, analyses of different manufacturing process of the tibial inserts, like net shaped molded components or sterilization procedures are missing in this context. The observations presented in this study firstly demonstrate alterations and damage of UHMWPE inlay primarily specific for the Genesis II condylar constrained design, even if there were case reports of the same problems for other designs using an inlay with a tibial post.

With this investigation we furthermore would like to point out that scanning electron microscopy enables a local inspection of surfaces. Therefore different types of defects like micro-fissures or scratches having a size far below 1 μm can be detected as well as be characterized. Obviously, this is a unique advantage over rather simple macroscopic methods like microabrasive wear testing of UHMWPE [23] in cases when induced modifications and damages, respectively, just sporadically occur in micro-regions of a surface.

## Conclusion

This investigation demonstrates that PE inlays of constrained total knee prostheses have a weak point at the zone where the post merges into the plateau. Already short periods of weight bearing cause significant damage inform of micro-fissures and clefts especially at the post corners and the posterior area. Particularly, in overweight patients as well as in patients with ligamentous insufficiency, a mechanically more solid PE-inlay is required. Furthermore, an extended examination of the patient after a constraint total knee arthroplasty with the symptoms of unknown discomfort, persistent effusion of the joint, instability or deviation of the leg axis should be performed because of a suspicion of a PE inlay failure.

Further investigations are needed to find out if lower acting loads can cause a tibial post breakage in polyethylene inlays with this demonstrated damage. Also the role of rotational forces and ligament insufficiencies must be evaluated in following studies.

## Abrevations

polyethylene: PE; scanning electron microscopy: SEM; secondary electrons: SE; total knee arthroplasty: TKA; ultra-high-molecular weight polyethylene: UHMWPE; nanometers: nm; micrometers: μm; kilo electron volt: keV.

## Competing interests

The authors declare that they have no competing interests.

## Authors' contributions

**AS **participated in the design of the study, collected patients' inlays and data, drafted the manuscript.** COT **participated in the design of the study, collected patients' inlays and data, drafted the manuscript and revised the manuscript for grammar and content.** RR **participated in the design of the study, performed the scanning electron microscopy with his assistants, helped to draft the manuscript and revised the manuscript for grammar and intellectual content.** SF–W **participated in the design of the study, was responsible for the organization of the study, helped to draft the manuscript and revised the manuscript for intellectual content.

## Pre-publication history

The pre-publication history for this paper can be accessed here:


